# Public Perceptions of Marriage Among Individuals with Bipolar Disorder Versus Schizophrenia: A Comparative Analysis

**DOI:** 10.1192/j.eurpsy.2025.2276

**Published:** 2025-08-26

**Authors:** Y. Trabelsi, B. N. Saguem, N. Jaafar

**Affiliations:** 1 Psychiatry Department, Farhat Hached Hospital, sousse, Tunisia

## Abstract

**Introduction:**

Marriage is widely regarded as an important part of social life, providing emotional support and stability. However, public perceptions of marriages involving individuals with psychiatric conditions like schizophrenia and bipolar disorder are not well understood. Stigma likely plays a crucial role in shaping these perceptions and affects societal attitudes toward such unions.

**Objectives:**

This study aimed to explore and compare public perceptions of marriage in individuals with schizophrenia and in individuals with bipolar disorder, and to examine how these views affect their marital prospects.

**Methods:**

A cross-sectional survey was conducted using an online form to gather data from the general population. It covered socio-demographic and clinical variables, as well as detailed descriptions of schizophrenia and bipolar disorder symptoms and outcomes. The survey also included questions on public attitudes toward marriage involving individuals with schizophrenia and bipolar disorder. Participants answered 13 questions about the right of individuals with these conditions to marry, their capacity to maintain a stable relationship, and whether they would personally consider or recommend marrying someone with these disorders. This study was inspired by the article of Kumar et al., 2019).

**Results:**

The study involved 304 participants, mostly young adults, with 246 being women. Around 35.6% had a family history of psychiatric illness, and 23.35% lived with someone with a psychiatric disorder. The findings revealed significant differences in public perceptions of marriage for individuals with schizophrenia versus bipolar disorder. Participants were more strongly opposed to marriage as a cure, especially for schizophrenia (p<10^-3), and more had never considered finding a partner for someone with schizophrenia compared to bipolar disorder (p<10^-3). Reluctance to marry someone with schizophrenia was also significantly higher, even if compatibility was present (p<10^-3).

Regarding bipolar disorder, concerns about marriage exacerbating symptoms were more prevalent compared to schizophrenia (p=0.001). Despite these concerns, the stigma around marriage with individuals affected by bipolar disorder appeared less severe, as indicated by participants’ greater openness toward the possibility of such a union in individuals with schizophrenia (p<10^-3).

**Conclusions:**

Results emphasize the need for targeted awareness and educational initiatives to address misunderstandings and support the marriage goals of individuals with mental health conditions. Future studies should examine the factors shaping these perceptions and develop approaches to promote a more inclusive perspective on marriage and mental health, tackling stigma and challenging widespread stereotypes.

**Disclosure of Interest:**

None Declared

